# Concentrating Model Solutions and Fruit Juices Using CO_2_ Hydrate Technology and Its Quantitative Effect on Phenols, Carotenoids, Vitamin C and Betanin

**DOI:** 10.3390/foods10030626

**Published:** 2021-03-16

**Authors:** Alexander Rudolph, Amna El-Mohamad, Christopher McHardy, Cornelia Rauh

**Affiliations:** Department of Food Biotechnology and Food Process Engineering, Technische Universität Berlin, Straße des 17. Juni 135, 10623 Berlin, Germany; amnaelmohamad@gmail.com (A.E.-M.); christopher.mchardy@tu-berlin.de (C.M.); cornelia.rauh@tu-berlin.de (C.R.)

**Keywords:** food processing, fruit juice concentration, gas hydrate, food quality, sustainability

## Abstract

Fruits have an important economic impact in the context of plant-based food production. The consumption of fruit juices, mostly produced from concentrates, is particularly noteworthy. Conventional concentration methods do not always enable a sustainable and gentle concentration. The innovative gas hydrate technology addresses this point with its energy-saving, gentle character, and high concentration potential. In this study, the concentration of fruit juices and model solutions using CO${}_{2}$ hydrate technology was investigated. To find a suitable operating point for hydrate formation in the used bubble column, the hydrate formation in a water–sucrose model solution was evaluated at different pressure and temperature combinations (1, 3, 5 °C and 32.5, 37.5, 40 bar). The degrees of concentration indicate that the bubble column reactor operates best at 37.5 bar and 3 °C. To investigate the gentle processing character of the hydrate technology, its quantitative effects on vitamin C, betanin, polyphenols, and carotenoids were analyzed in the produced concentrates and hydrates via HPLC and UV/VIS spectrophotometry. The results for fruit juices and model solutions imply that all examined substances are accumulated in the concentrate, while only small amounts remain in the hydrate. These amounts can be related to an inefficient separation process.

## 1. Introduction

Consumers are increasingly aware of the importance of healthy and plant-based foods. Among these foods, fruit juices enjoy great popularity. They are considered to be both natural and healthy. Health benefits are directly related to phytochemicals, such as polyphenols or carotenoids, which are present in fruits [1]. For this reason, juices experience high demand in the market. Across geographic regions in 2010, 0.66 to 0.013 servings/day were consumed worldwide while the global market revenue was around $111 billion in 2014 with an annual growth of 3% between 2010 and 2014 [2,3]. In 2018, 35.9 billion L of fruit juice and nectar were consumed worldwide, with 9.1 billion L being consumed in the EU [4] and 2.3 billion L consumed in Germany [5]. Large quantities of fruit juices are concentrated during production. Thereby, further processing and storage properties are improved, while, at the same time, logistical advantages open up, as costs for packaging, storage, and transport can be saved [6,7]. 30.35 billion L of the global fruit juice consumption were related to powdered and concentrated juices in 2018 [8]. In the EU, 3.8 billion L of the consumed juices were produced from concentrates in 2018 [9], while the corresponding value for Germany was 1.39 billion L in 2014 [10].

Removing water using conventional methods involves a high level of equipment and energy consumption. In many cases, evaporation is used to concentrate juices, since a degree of concentration up to 85% can be reached [7]. Because of the prevailing temperatures of at least 70 °C [6], 180 to 2160 kJ/kg water are required to remove water from juices [11]. Besides, the process impairs product quality, because heat-sensitive substances, like vitamins or polyphenols, are destroyed or altered [6,11,12]. Moreover, recovery systems are used to maintain product quality concerning volatile compounds, such as aroma [13]. Freeze concentration is available as an alternative to avoid the loss or damage of heat-sensitive or volatile substances [6,7]. It is based on a multistage crystallization of water to ice and its separation (e.g., by centrifuges). Thereby, a degree of concentration of up to 55% can be reached [7,11]. The typical energy requirement of 936–1800 kJ/kg water in this process is less than the amount of energy that is required for evaporation [11]. Nevertheless, there is still a need to improve conventional concentration methods for sustainability and energy efficiency reasons.

The need for a resource- and product-saving process has brought gas hydrate technology into the focus of industrial and scientific research. Gas hydrates were discovered in the early 19th century by Sir Humphry Davy, but their first industrial relevance came in 1934 when Hammerschmidt discovered that hydrates were responsible for blocking gas pipelines [14]. More recent scientific studies concern carbon capture and storage [15,16,17,18,19], water withdrawal from ionic liquids [20], and seawater desalination [21,22,23,24]. Moreover, hydrate technology is used in the food sector, as it provides an innovative approach to concentrate juices with low energy requirements of 252–360 kJ/kg water [11]. The conditions for hydrate formation are in the range of low temperatures (below 27 °C) and moderate pressures (above 6 bar), depending on the process gas [25,26,27]. CO${}_{2}$ hydrate forms between 30 bar and 80 bar and from 1 °C to 8 °C [11]. This combination of temperature and pressure enables gentle and sustainable processing. So far, the concentration of liquid foodstuffs using gas hydrate technology has been carried out on a laboratory scale with fruit and vegetable juices or coffee [11,13,28,29,30,31,32,33]. The main focus of the studies concerning juices was either on hydrate formation kinetics and equilibrium conditions or achievable degrees of concentration, indicating the need for new data, especially on the preservation of valuable fruit juice components. It was possible to concentrate apple juice and orange juice in a bubble column reactor to values between 20 °Brix and 27 °Brix at 37 bar and 2.5 °C with a yield of 40% [11]. Furthermore, the influence of pressures between 30 bar and 45 bar was analyzed in more detail. From 30 bar to 37 bar and 42.5 bar to 45 bar produced concentrates had sugar contents from 12 °Brix to 15.5 °Brix, whereas, at 40 bar, concentrates showed values of 20 °Brix [32]. Besides temperature and pressure, the ratio of sample volume to reactor volume directly influences the hydrate formation. For a bubble column configuration, the optimum ratio was found between 33% and 40% [11,32]. For stirred tanks, it has been reported that the ratio is between 33% and 35% [13,28,29]. Concentrates with over 40 °Brix could be produced with this ratio in stirred tanks at 1 °C to 2 °C and 35 bar [13].

The results reported in the literature highlight the importance of choosing the optimal temperatures and pressures. However, hydrate formation conditions for juices are shifted towards lower temperatures and higher pressures as compared to CO${}_{2}$-water systems due to inhibitory effects of food ingredients [29,31,32,33]. Especially, sugars, such as sucrose, fructose, and glucose, are known for inducing this shift [34], because they are the major components in juices. Nevertheless, the presented studies imply that concentrating juices using CO${}_{2}$ hydrate technology is promising, even though further process optimization is required to reach the required degree of concentration for industrial applications. Moreover, there is a need for studies evaluating the concentrate quality, since no parameters concerning the preservation of valuable fruit juice compounds have been evaluated so far. Data that were collected on this topic for the first time would contribute to underlining the advantage of the innovative gas hydrate technology over conventional concentration methods. Therefore, several valuable juice components have to be investigated to show that product quality is not impaired during fruit juice concentration using gas hydrate technology. To estimate the preservation of fruit juice compounds, such as polyphenols, carotenoids, or vitamins, knowledge of hydrate formation and structure is indispensable. The basic principle of gas hydrate technology is based on water molecules (host molecules) forming an ice-like cage structure in whose cavities guest molecules with low molecular weight are trapped [14]. Water builds up the cage structure, while the guest molecules stabilize the gas hydrate by van der Waals forces [35]. The thermodynamically preferred crystal structure will form, depending on the combination of temperature, pressure, and the availability of hydrate-forming guest components [14]. To this day, three crystal structures sI, sII, and sH have been identified [14,26,27,36]. CO${}_{2}$ as a food-safe gas forms sI hydrates [35]. Water-soluble acid gases, hydrophobic components, water-soluble polar components, and water-soluble alkylammonium salts are other known types of guest molecules in hydrate structures [37]. The main aspects defining whether a substance can participate in hydrate formation are its shape, size, and chemical nature [35,38].

Hydrate formation in technical systems is governed by the reaction and transport kinetics as well as the equilibrium thermodynamics. The driving force is proportional to the distance of the prevailing temperature and pressure conditions from the equilibrium state [39]. The main principle to induce hydrate formation is to inject gas into an aqueous phase or water into a gaseous phase. CO${}_{2}$ could be even liquid, as described for the formation of thin CO${}_{2}$ hydrate films referring to oceans [40]. For several applications, reactors must ensure the efficient removal of heat during the hydrate formation [41]. This is achieved by improving heat transfer by mechanical mixing in stirred tanks or bubble towers [42]. At the laboratory scale, stirred tanks are the most used reactor types [43]. A disadvantage of stirred tanks is that the higher the volume fraction of hydrate becomes more energy is needed to reach the required degree of turbulence due to the increased viscosity of the hydrate slurry [42]. Therefore, bubble columns have been introduced as one alternative. In bubble columns, gas hydrate directly forms around the bubble at the gas–liquid interface [44]. Any further formation is hindered by additional resistance for interphase mass transport that was caused by the hydrate shell [42,45]. Larger interface areas have to be created by either reducing the bubble sizes or increasing the gas velocity to overcome this issue or shockwaves could be used to crack the shells to renew the reaction sites [42,44,45,46]. For this reason, the gas hydrate process cannot only be based on the equilibrium conditions, but also on the conditions in the reactor.

The preservation of quality is considered to be a great advantage of gas hydrate technology. Therefore, this research mainly aims to analyze whether polyphenols, carotenoids, vitamin C, and betanin are participating in hydrate formation within fruit juices and a water-sucrose-betanin model solution for the first time. Consistent with the knowledge regarding gas hydrate structures, no incorporation of valuable substances into the hydrate is expected. According to the literature, Brix values for concentrates produced by CO${}_{2}$ gas hydrate in bubble columns vary depending on pressure and temperature. Therefore, in this study, a water-sucrose model solution representing juices is used to define a specific working point in a bubble column reactor because mass and energy transport will influence the hydrate formation and thus the concentration process. Thereby, possible interactions in the complex juice matrix are eliminated, and the hydrate formation is expected to be more reproducible. Although the used solution is a simplified model matrix, sugars are the main components of juices. Thus, the model solution is stated to be an adequate replacement for juices.

## 2. Materials and Methods

### 2.1. Materials

A water-sucrose model solution (10 °Brix) was used to identify a suitable temperature and pressure combination for the hydrate formation within the bubble column reactor. After the operating point had been identified, a water-sucrose-betanin model solution with 10 °Brix and 1 g/L betanin was used for further experiments. Its purpose was to investigate whether betanin is going to be part of the hydrate structure. Furthermore, the colored solution made it possible to examine optically if the hydrate contains the dye. This shows how efficient the separation of hydrate and concentrate is working. In a third series of experiments, clear apple juice, cloudy apple juice, and orange juice were used to test whether polyphenols, carotenoids, or vitamin C are incorporated into the hydrate structure. The juices were purchased from local supermarkets. Table 1 provides an overview of all used media, the analyzed substances, the investigation aim, and the number of experimental repetitions. In all experiments, CO${}_{2}$ hydrate was formed with high purity CO${}_{2}$ (99.95%, Air Liquide, Paris, France).

### 2.2. Reactor System

**Figure 1:**
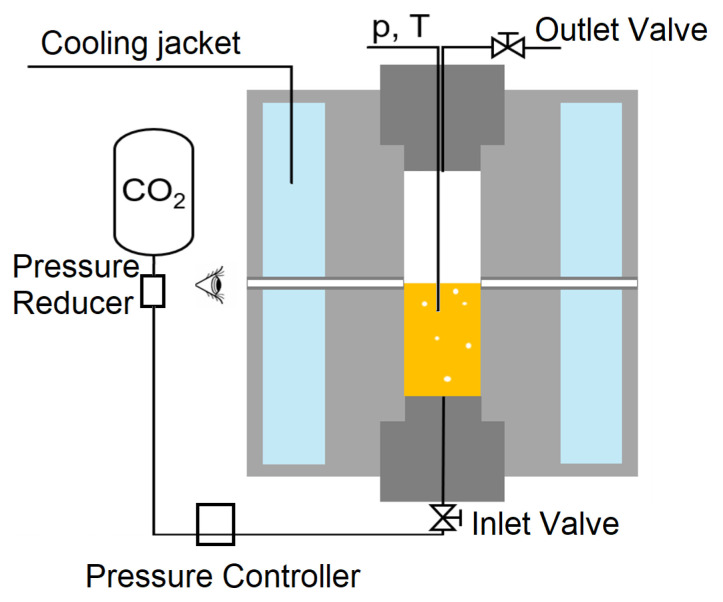
Schematic of the used bubble column reactor.

The high-pressure reactor that is shown in Figure 1 is designed to withstand pressures of up to 5000 bar. It has a volume of 1.5 L and was operated as a bubble column.

To control and measure the pressure, a pressure controller (SLA5810, Brooks Instrument, Dresden, Germany) and a pressure sensor (DRTR-AL-10V-R100B, B+B Thermo-Technik GmbH, Donaueschingen, Germany) were used. A cooling jacket with a circulating chiller (L002326 Proline RP 855, Lauda, Lauda-Königshofen, Germany) realized the cooling of the system, while the temperature within the reactor was measured with a thermocouple. Thus, the conditions that are needed for gas hydrate formation were measured, controlled, and recorded (OMB-DAQ-2408, OMEGA Engineering, Deckenpfronn, Germany).

### 2.3. Experimental Procedure

To find a suitable operating point, experiments at different levels of temperature (1 °C, 3 °C and 5 °C) and pressure (32.5 bar, 37.5 bar and 40 bar) were performed and the formation of gas hydrate in the water–sucrose model solution was evaluated for each temperature–pressure combination. Similar conditions are reported in the literature for concentrating juices using gas hydrate technology [11,13,32]. The hydrate formation in the water–sucrose–betanin model solution and the three juices for the quality analysis was investigated at 37.5 bar and 3 °C.

**Figure 2:**
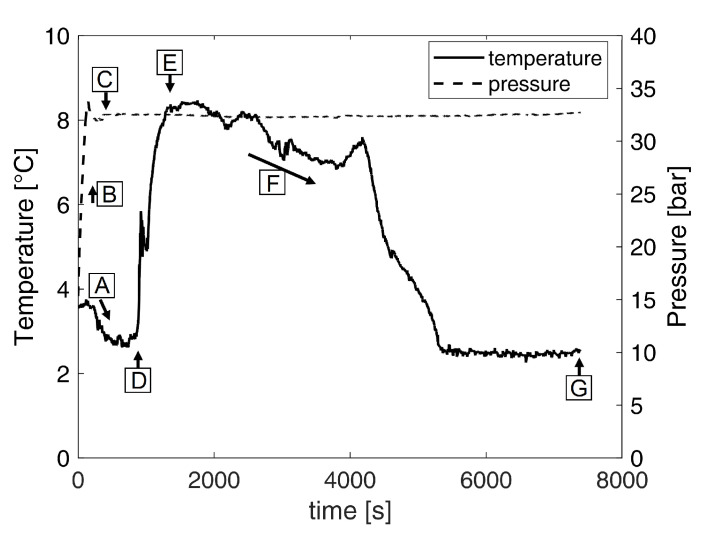
Temperature and pressure curves are exemplarily shown for hydrate formation in the water-sucrose model solution at 32.5 bar and 3 °C: the system is cooled to the desired temperature (**A**). Subsequently, pressure is build up (**B**) and set to the desired value (**C**). The start of hydrate formation is indicated by a rising temperature (**D** to **E**). This temperature rise is followed by another cooling step (**F**). The experiments were stopped two hours after the start of hydrate formation (**G**).

For all experiments, the reactor was first cleaned with hot water at a temperature of 100 °C. After cleaning, the interior of the reactor was dried with a cloth to remove any foreign particles. 550 mL juice or model solution was then filled into the reactor. This corresponds to a sample to reactor volume ratio of about 36%, which is in the suitable range of 33% to 40% for bubble columns that were reported in literature [11,32]. Shortly before cooling to the desired temperature for a specific experiment, the system was flushed with CO${}_{2}$. Thereby, air was removed and pressure built up in the reactor. Subsequently, the pressure was set to the desired value by an automatic adjustment of the inlet gas flow. Figure 2 shows the typical pressure and temperature curves during an experiment with the water–sucrose model solution at 32.5 bar and 3 °C. To start the process, the outlet valve was opened. The start of hydrate formation was indicated by a rising temperature within the reactor, which is related to the exothermal character of hydrate formation (see Figure 2). The experiments were stopped two hours after the start of hydrate formation to guarantee a solid hydrate structure.

After the hydrate had formed, it was removed from the reactor as a solid block. Claßen et al. (2020) [13] describe that the use of a pellet press and an additional washing step can improve the separation of the hydrate and concentrate. They advise that the separation should take place under hydrate stable conditions for sugar contents above 30 °Brix. This separation concept was adapted from seawater desalination [21]. Because around 20% to 40% of the hydrate was adhering concentrate in the present study, the gas hydrate was pressed by a wire press for 60 s applying a pressure of 450 bar to ensure a separation of the phases. The hydrate is metastable under the pressing conditions and, consequently, a dissociation of the hydrate is induced. However, the time scale of the pressing procedure was short enough to prevent the hydrate from excessive dissociation. After the separation, the hydrate was carefully dissociated in a microwave in a maximum of two to three time steps of 10 to 15 s, depending on the amount of hydrate. Finally, the concentrate, the dissociated hydrate, and the drained liquid phase obtained from the pressed hydrate were analyzed for total phenolics, total carotenoids, vitamin C, or betanin, depending on the initial solution according to Table 1.

### 2.4. Sugar

The sugar content in °Brix of the samples was determined in triplicate without any further preparation in an oscillating U-tube (DMA 4500M, Anton Paar, Graz, Austria) and a digital refractometer (RFM 80, Bellingham + Stanley, Xylem Analytics, Weilheim, Germany). For calculating a yield, the mass of the concentrate, the hydrate, and the drained liquid pressed from the hydrate were measured once. Equation (3) in Section 3.2 presents the corresponding calculation of the yield.

### 2.5. Betanin

The betanin content in the samples was photometrically evaluated at 538 nm (Lambda 25 UV/VIS Spectrometer, Perkin Elmer, Rodgau, Germany), and the betanin concentration was then calculated from a calibration line. The measurements were conducted in duplicate. For the determination of the calibration line 0.375 g, 0.75 g, 1 g, 1.5 g, 2.25 g, and 3 g of betanin (AB137484, abcr GmbH, Karlsruhe, Germany) were dissolved in 1 L distilled water. These solutions were measured at 538 nm against a blank of pure distilled water. The calibration line was determined twice with a triplicate determination on each data point. Furthermore, a calibration line with a 10 °Brix solution and the betanin concentrations given above was produced. The added sucrose had no effects on the calibration line.

### 2.6. Total Phenolics

One of the most used methods for determining the total phenolics is the Folin–Ciocalteu assay [47]. The procedure used in this research is based on the publication of Singleton, Orthofer, and Lamuela-Raventós (1994) [48], with some modifications. 200 μL of diluted samples was obtained from the concentration process of clear apple juice and orange juice (1:100 with distilled water) or distilled water as a blank was mixed with 1 mL of diluted Folin–Ciocalteau reagent (Sigma-Aldrich, Merck, Darmstadt, Germany, diluted 1:10 with distilled water) in triplicate. After 30 s, 800 μL of 7.5% Na${}_{2}$CO${}_{3}$ solution were added. All of the samples were then incubated at 40 °C for 30 min., shaken, and subsequently measured in the spectrophotometer at 765 nm. Finally, the total phenolics were calculated from a gallic acid calibration line.

### 2.7. Total Carotenoids

Oranges tend to contain more carotenoids than apples, and the carotenoids are also present in orange juice [49]. Therefore, only orange juice was considered in the analysis of total carotenoids. The method was taken from a DIN EN standard [50]. 50 mL of each orange juice sample were analyzed for total carotenoids in triplicate. The carotenoids were precipitated with 1 mL Carrez-I and Carrez-II solution, respectively. Subsequently, the carotenoids were extracted from the precipitate with acetone and transferred to petroleum ether. The total carotenoid content was spectrophotometrically determined against a blank consisting of pure petroleum ether at a wavelength of 450 nm. The total carotenoids are expressed as β-carotene equivalents $\rho (C_{40}H_{56})$, according to the DIN EN standard [50]:

(1) $$\rho \left(C_{40}H_{56}\right)=A\cdot 4.00\cdot \frac{V_{2}}{V_{1}}.$$

In Equation (1), *A* is the extinction of the petroleum ether extract, 4.00 represents a mean conversion coefficient, $V_{2}$ is the volume of the petroleum ether extract, and $V_{1}$ is the initial volume of the analyzed sample.

### 2.8. Vitamin C

The vitamin C content was measured by HPLC (Ultimate 3000, Thermo Fisher Scientific, Waltham, MA, USA) with a hyperchrome HPLC column. The temperature of the HPLC column oven (TCC-3000SD) was 25 °C. The mobile phase was isocratic with 10 mM sulfuric acid (pH-value of 2.2). The method used was developed following the publications of Rückemann (1980) [51] and Sood et al. (1976) [52]. 25 mg of L-ascorbic acid (purity 99%, Sigma–Aldrich, Merck, Darmstadt, Germany) were dissolved in 25 mL of 3% meta-phosphoric acid and further diluted in 3% meta-phosphoric acid to produce a standard solution. All the phases from the concentration process of orange juice, clear apple juice, cloudy apple juice, and the initial solutions of the respective juices were frozen until the analysis. The samples were then filtered and the filtrate was used for HPLC analysis. Therefore, all of the samples were mixed 1:10 with 3% meta-phosphoric acid to stabilize the vitamin C. The samples and standard solution were pressed through an HPLC syringe filter to remove coarser particles and prevent membrane clogging. A defined volume of 20 μL of the sample was injected into the dosing loop. In the separation column, the samples were analyzed by the detector (diode array UV detector, DAD-3000) at 254 nm. The measurements of the samples were carried out in duplicate.

### 2.9. Statistical Data Evaluation

For all experiments, the mean values $\bar{x}$ and the standard errors of the mean $\sigma_{\bar{x}}$ were calculated. The standard error of the mean is defined as:

(2) $$\sigma_{\bar{x}}=\frac{s}{\sqrt{n}}.$$

In Equation (2), *s* represents the standard deviation and *n* is the number of samples. The mean of the Brix values of the hydrate formation repetitions was determined for identifying the working point. Within the sugar content measurements, the standard error of the mean is approximately zero. Consequently, the standard error of the mean between the repetitions of hydrate formation (n=3) is considered to be the main error occurring during this experiment and, thus, it is presented in the figures of Section 3.2. Because the masses needed for the calculation of the yield were measured once, the calculated mean is the mean of the hydrate formation repetitions. The standard error of the mean represents the standard error between these repetitions (n=3).

The mean of the data that were obtained from the quality analyses was calculated from all determinations of one analysis (e.g., total phenolics) and all respective repetitions of hydrate formation. For example, for total phenolics, this would take the three repetitions of the hydrate formation and the triple determination of the Folin–Ciocalteu assay into account (n=9). The standard error of the mean of these values is finally calculated. For the determination of total carotenoids the number of samples is nine (n=9), for vitamin C four (n=4), and for betanin six (n=6).

## 3. Results

### 3.1. Hydrate Structure

The hydrate produced in the bubble column reactor has a different consistency, depending on the temperature and pressure conditions used.

**Figure 3:**
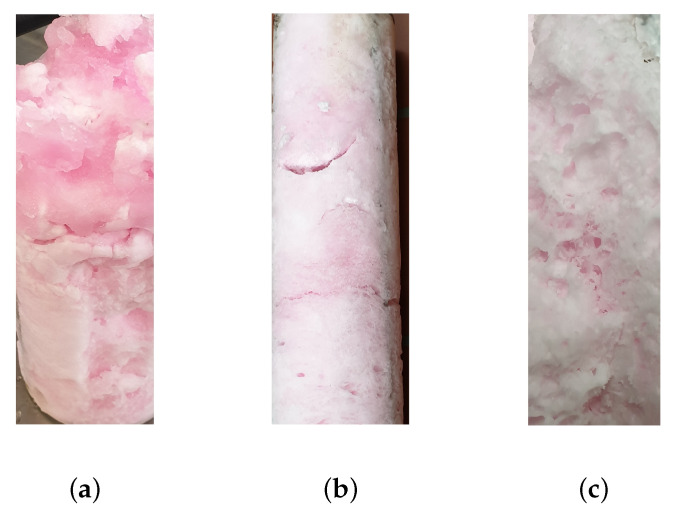
CO${}_{2}$ hydrate formed in the water-sucrose-betanin model solution at different temperature and pressure conditions using a bubble column reactor: (**a**) hydrate block with hydrate slurry formed at 40 bar and 1 °C, (**b**) solid hydrate block formed at 37.5 bar and 3 °C, and (**c**) porous hydrate structure formed at 37.5 bar and 3 °C.

Figure 3a depicts that at 40 bar and 1 °C a hydrate consisting of a solid block and hydrate slurry is formed in the water-sucrose-betanin model solution. At 37.5 bar and 3 °C, a solid and porous block forms from the same initial solution as shown in Figure 3b. This block fills almost the entire reactor volume of 1.5 L. The structure of the hydrate formed at 37.5 bar and 3 °C is porous, and Figure 3b, as well as Figure 3c, show that concentrate either adheres to the surface of the hydrate block or the porous structure. This underlines the need for a suitable separation of hydrate and concentrate.

### 3.2. Identification of the Operating Point

In bubble columns, CO${}_{2}$ hydrate is formed in fruit juices between 30 bar and 42 bar at temperatures between 1 °C and 10 °C [11,32]. Because of the shift in equilibrium conditions towards higher pressures and lower temperatures, corresponding values can be expected for the water–sucrose model solution [34].

**Figure 4:**
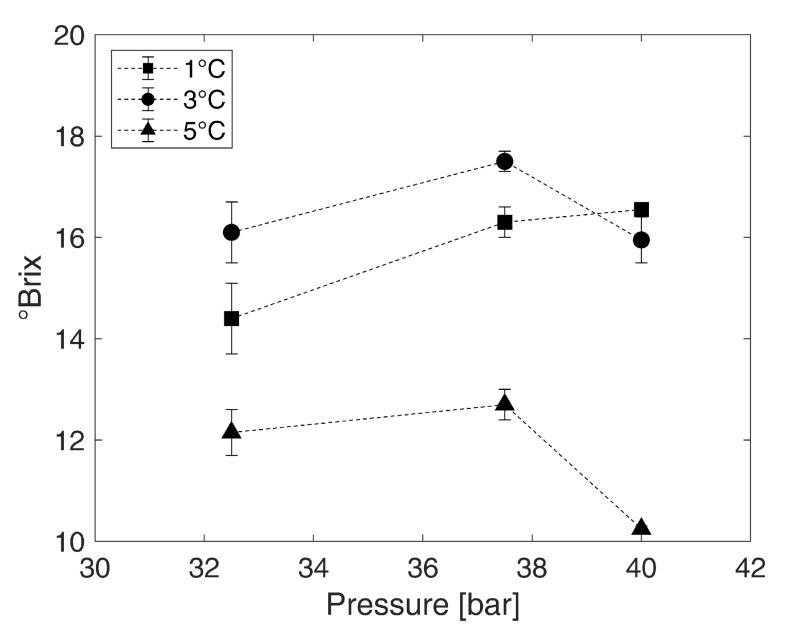
Reached sucrose content in °Brix in the concentrate produced from the water-sucrose model solution (10 °Brix) using CO${}_{2}$ hydrate technology at pressures of 32.5 bar, 37.5 bar, and 40 bar combined with temperatures of 1 °C, 3 °C, and 5 °C (error bars: standard error of the mean according to Section 2.9, the standard errors of the mean are given in Table S2).

The collected results in Figure 4 show that, for the tested pressure and temperature combinations, concentrates with Brix values from around 10 °Brix to nearly 18 °Brix can be produced from an initial solution with 10 °Brix. In the literature, values between 12 °Brix and 27 °Brix were reported for fruit juices [11,32]. Within all experiments of the present study, the Brix value of the hydrate phases was between 2 °Brix and 6 °Brix. At a pressure of 37.5 bar and a temperature of 3 °C, the best possible concentration in the used reactor system was achieved. Values of nearly 18 °Brix are reached, which corresponds to a concentration factor of about 1.8. At the same temperature, pressures of 32.5 bar and 40 bar lead to a slightly less satisfactory concentration by a factor of 1.6. If a temperature of 1 °C is used in the process, 14 °Brix to 16.6 °Brix are achieved at the same pressure levels. At 32.5 bar, the lowest value is obtained in the resolved range, but the achievable concentration increases at higher pressure. However, the difference between 37.5 bar and 40 bar is no longer significant and, thus, the influence of pressure decreases. The concentration process is least efficient at 5 °C. While, at this temperature and 37.5 bar, about 13 °Brix are still achieved and the Brix value for 32.5 bar is slightly above 12 °Brix. At a combination of 5 °C and 40 bar, nearly no concentration took place, since the sucrose content of the concentrate does not differ significantly from the starting solution in its sucrose content of 10 °Brix. The calculated standard errors of the mean are in the range of 0.05 and 0.7 °Brix for all samples. This indicates a high reproducibility of the hydrate formation.

The amount of the produced concentrate was in the further focus of the performed experiments. In order to determine the concentration yield for the used pressure and temperature conditions, the quotient of the achieved concentrate mass $m_{c}$, and the mass of the initial solution $m_{0}$ was determined:

(3) $$Yield\left[\%\right]=\frac{m_{c}}{m_{0}}\cdot 100\%.$$

**Figure 5:**
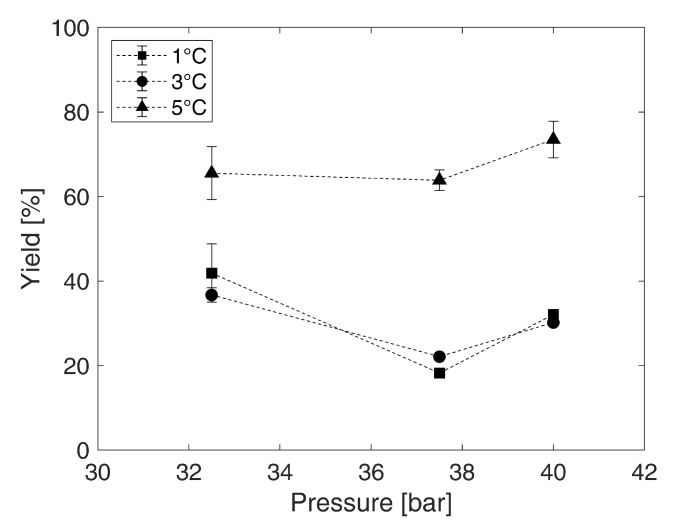
Reached yield in % expressed as a quotient of the reached concentrate mass $m_{c}$ after the concentration using CO${}_{2}$ hydrate technology and the mass $m_{0}$ of the initial water-sucrose model solution (10 °Brix) at pressures of 32.5 bar, 37.5 bar, and 40 bar combined with temperatures of 1 °C, 3 °C, and 5 °C (error bars: standard error of the mean according to Section 2.9, the standard errors of the mean are given in Table S2).

Figure 5 visualizes the yields for every analyzed temperature and pressure combination. The calculated standard errors of the mean are between 0.3 and 6.9% for all yields.

At 5 °C from 32.5 bar to 37.5 bar, the yield is nearly at 65%, whereas, at 5 °C and 40 bar, nearly 75% are reached. The yields at 1 °C and 3 °C are in the same range. At 32.5 bar, between 37% (3 °C) and 42% (1 °C) of the initial mass are present as concentrate after the process. If a pressure of 37.5 bar is used to produce a concentrate from the solution, the yield is between 18% to 22%, while at 40 bar yields of 30% to 32% were achieved. In comparison, the literature reports values of 40% for the concentration in a bubble column configuration [11]. From the data in the present research, it appears that a higher concentration results in a lower yield, since the values approximately directly correspond to the sucrose concentrations of the concentrate. This can be attributed to the fact that a lower amount of water is present in a higher concentrated concentrate.

### 3.3. Effect of the Concentration Using Gas Hydrate Technology on Valuable Ingredients

Because CO${}_{2}$ hydrate formation requires gentle processing conditions, valuable juice ingredients should not be negatively affected during the concentration of fruit juices and the water–sucrose–betanin model solution. Therefore, the quality experiments aimed to assess the preservation of betanin, polyphenols, carotenoids, and vitamin C.

**Figure 6:**
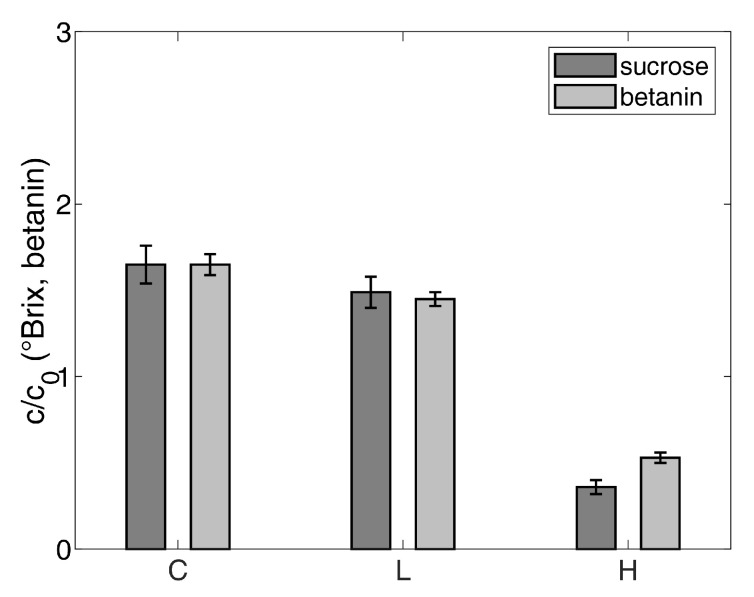
Quotients of the reached concentrations *c* after hydrate formation in the water-sucrose-betanin model solution and the initial concentrations $c_{0}$, respectively, for sucrose and betanin; C: concentrate, L: drained liquid from the hydrate, H: hydrate (error bars: standard error of the mean according to Section 2.9, the standard errors of the mean are given in Table S3).

Quotients of the achieved concentration *c* and the initial concentration within the water–sucrose–betanin model solution $c_{0}$ of sucrose and betanin, in each phase (concentrate, drained liquid pressed from the hydrate, hydrate), respectively, are presented in Figure 6. Thus, every value can be considered as a concentration factor and quotients above 1 indicate that a concentration took place. The absolute values of the betanin contents can be found in Table S1 of the Supplementary Material. For the water–sucrose–betanin model solution, the data show that the calculated quotients for sucrose and betanin in the concentrate are 1.65 and around 1.5 for the drained liquid from the pressed hydrate. Consequently, both sucrose and betanin are concentrated. Because the values for the drained liquid pressed from the hydrate are in the same range, it is assumed that the drained liquid is adhering concentrate. Nevertheless, sucrose and betanin are both found in the hydrate phase, but the calculated quotient for betanin is 0.5, whereas, for sucrose, it is 0.4. This means that more betanin than sugar is present in the hydrate phase. The standard errors of the mean are between 0.03 and 0.11 for all data presented in Figure 6.

**Figure 7:**
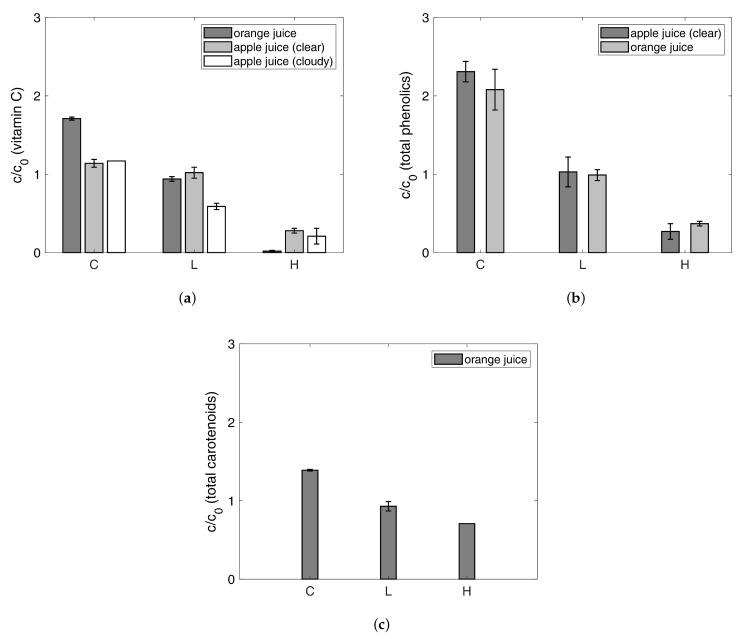
Quotients of the reached concentrations c produced and the corresponding initial concentrations $c_{0}$ for all phases from the concentration process: (**a**) vitamin C for orange juice, apple juice (clear) and apple juice (cloudy), (**b**) total phenolics for orange juice and apple juice (clear), and (**c**) total carotenoids for orange juice. C: concentrate, L: drained liquid from the hydrate, H: hydrate (error bars: standard error of the mean according to Section 2.9, the standard errors of the mean are given in Table S3).

Figure 7a shows the results of the vitamin C measurements. Being analogous to the betanin determinations, the quotient *c*/$c_{0}$ was calculated to obtain a measure of concentration. The standard errors of the mean are between 0.001 and 0.1 for all vitamin C data. The results indicate that the highest amounts of vitamin C are found in the concentrates of orange juice and both apple juices. During juice concentration using gas hydrate technology, the vitamin is concentrated by a factor of about 1.7 for orange juice. In both apple juices, the concentration factors for vitamin C are around 1.2. For all samples, the concentration ratios in the drained liquids pressed from the hydrate are between 0.5 to 1. It is noticeable that the gas hydrate contains only small amounts of vitamin C (maximum quotients of 0.3). The values for the hydrate phase produced in both apple juices differ from the values for orange juice. The absolute amounts of vitamin C in orange juice are much higher than the absolute amounts of vitamin C in apple juice (both clear and cloudy). In the hydrate phase, these values are even smaller (see Table S1). Because the data presented in Figure 7 are relative, even small differences in the absolute values of vitamin C content in the apple juices have a great impact on the presented data. The results on vitamin C imply that separating the gas hydrate and adhering concentrate has to be further improved.

The results regarding total phenolics for the concentration of clear apple juice and orange juice using gas hydrate technology that are shown in Figure 7b underline the inefficient separation. The quotient *c*/$c_{0}$ was determined, as before. The standard errors of the mean for total phenolics are between 0.03 and 0.3. Polyphenols are concentrated in the concentrate by more than factor 2 via CO${}_{2}$ hydrate formation. Again, quotients around 1 are found regarding the liquids from the pressed hydrate. The hydrate phases contain the smallest amount of polyphenols. For clear apple juice, this value is at 0.3 and for orange juice at 0.4. Figure 7c visualizes the results for the quotient *c*/$c_{0}$ concerning total carotenoids in all the phases gained during the concentration of orange juice. The standard errors of the mean for total carotenoids are between 0.003 and 0.06. Most of the carotenoids are part of the concentrate, which is indicated by the concentration factor of 1.4. A value below 1 is found regarding the drained liquid from the pressed hydrate. The hydrate phases contain just slightly fewer carotenoids with a quotient of 0.7.

All of the results indicate that polyphenols, carotenoids and vitamin C are concentrated by a factor of 1.2 to more than 2 within the juice during the gas hydrate process. The factors for the hydrate phase are 0.7 and below. Although only small amounts remain in the hydrate, the separation of the hydrate and the adhering concentrate is not efficient. Especially, carotenoids are not separated sufficiently by pressing. For the support of the presented data, the absolute values corresponding to the relative data that are presented in Figure 7 can be found in Table S1 of the Supplementary Material.

## 4. Discussion

### 4.1. Identification of the Operating Point

Overall, the results regarding the operation point highlight the effect of pressure on the concentration process. From 32.5 bar to 37.5 bar, the concentration of the water–sucrose model solution at temperatures of 1 °C, 3 °C and 5 °C improves. Beyond these pressures, the concentration process does not get more efficient or even deteriorates. This effect is described for fruit juices in literature as pressures below 37 bar or 40 bar lead to a decreased concentration efficiency as well as pressures beyond these values [32]. For the effect of different temperatures, the results indicate that temperatures of 1 °C and 3 °C should be used instead of 5 °C. According to the literature, a temperature of 2.5 °C is suitable for concentrating juices in a bubble column reactor [11]. In the present study, between 1 °C and 3 °C, the reached sugar contents in the hydrate are close, which implies that lowering the temperature is just applicable to a certain degree. Because hydrate technology is stated to consume less energy than conventional concentration methods [11] cooling to 3 °C saves energy as compared to the processing at 1 °C. Thus, pressure and temperature are both limiting factors for the concentration process. This limiting influence is the reason that nearly no concentration took place at 5 °C and 40 bar. The thermodynamic driving force is proportional to the distance of the working point to the equilibrium conditions [39]. All of the results indicate that high pressures can reduce concentration efficiency. At 40 bar, the concentration process is no longer efficient and in combination with the reduced driving force due to the temperature of 5 °C the hydrate formation is hindered. Thus, at this temperature and pressure combination, the concentration process is not taking place, since nearly no hydrate is formed. Furthermore, the state of aggregation of CO${}_{2}$ influences the concentration process in the bubble column at pressure levels above 37.5 bar and temperatures of 1 °C or 3 °C. At these conditions, CO${}_{2}$ is liquid, and Shindo et al. (1993) [40] assume that a formation of thin hydrate films takes place in liquid CO${}_{2}$. However, the results that are presented in this research imply a decrease in concentration efficiency if liquid CO${}_{2}$ is present. Liquid CO${}_{2}$ changes the reaction and transport kinetics in the bubble column, which might directly affect the hydrate formation. Consequently, for a better understanding of every detail, kinetic and thermodynamic studies should be conducted and connected to this research.

Concerning the maximum achievable sugar concentration in the concentrate produced in bubble columns by hydrate formation, the values for fruit juices in the literature are higher with 20 °Brix to 27 °Brix [11,32], as compared to nearly 18 °Brix reached in this study for the water–sucrose model solution. This can be related to the complexity of the fruit juice matrix. Inhibitory ingredients in fruit juices and sugar solutions affect hydrate formation by shifting the hydrate formation conditions towards higher pressures and lower temperatures [29,31,32,33,34]. The best formation conditions in the present study for the water–sucrose model solution and juices reported in the literature [11,32] are in the same range. Therefore, it can be assumed that hydrate formation in juices is strongly influenced by the effect of sucrose. Because the formation conditions are very similar, the concentration process is further affected by either process kinetics or other components of the juices that might promote hydrate formation. Besides the need for additional data on kinetics and thermodynamics concerning the concentration process, further studies on the effect of fruit juice components or components of liquid foodstuff, in general, should be conducted.

For an industrial application, high sugar contents within the concentrate are needed. The reached sugar contents in the bubble column reactor during the concentration of the water-sucrose model solution of nearly 18 °Brix are much lower than over 40 °Brix, which can be achieved in stirred tanks for fruit juices in literature [13]. The results imply that further hydrate formation is hindered in the bubble column reactor used in the present study. According to the literature, the hydrate shell can negatively influence the hydrate formation [42,45]. Additional to the hydrate shell, inhomogeneities of the mass and energy distributions can be expected in the bubble column reactor. Thus, a better mixing, as achieved in a stirred tank, might help to improve the concentration process. Because the reactor contains more hydrate with increasing hydrate formation, solid block structures form almost throughout the entire bubble column reactor (see Figure 3b). In a stirred tank, shear forces break up hydrate blocks, leading to a slurry-like consistency. Consequently, the energy requirements of stirred tanks are higher. Nevertheless, the break-up of large hydrate structures improves further hydrate formation, which makes higher degrees of concentration possible.

### 4.2. Effect of the Concentration Using Gas Hydrate Technology on Valuable Ingredients

The results of this study indicate that polyphenols, carotenoids, vitamin C, and betanin are concentrated during the concentration of fruit juices or a water-sucrose-betanin model solution using gas hydrate technology. For all of the examined substances, the concentration factors are between 1.2 to 2.2 in one concentration step. Only small amounts remain in the hydrate phase. Based on the present results, no conclusions can be made regarding the mechanisms behind the incorporation of polyphenols, carotenoids, vitamin C or betanin, as further research is needed. However, the dissociation enthalpies of fruit juices and pure CO${}_{2}$-water systems that are calculated in the literature are in the same range. CO${}_{2}$-water systems have a dissociation enthalpy of 85.19 kJ/mol, and the dissociation enthalpies of orange and apple juice are 85.32 kJ/mol and 86.64 kJ/mol, respectively [32]. Thus, the produced gas hydrate only should consist of water and CO${}_{2}$. A reason for the preservation of valuable substances is the hydrate structure itself. Referring to the literature, CO${}_{2}$ hydrate forms a sI structure consisting of cavities that only allow for molecules of a certain size and a low molecular weight to be part of the hydrate [14]. The maximum guest molecule size is about 9 Å in sH structure, whereas molecules with a size of around 4 Å to 7 Å form sI or sII hydrates [35]. Typical substances that are part of sI hydrates are CO${}_{2}$, ethane, and methane [25]. Furthermore, the guest molecule must not contain either a single strong hydrogen-bond group or several moderately strong hydrogen-bonding groups [38]. Because vitamin C, polyphenols, and carotenoids are either large molecules or molecules with hydroxyl groups, their size and chemical nature do not allow them to be part of the hydrate structure. Consequently, the reason for the remaining amount of these substances in the hydrate phase is probably not related to hydrate formation. Much more likely is an influence of processing, especially concerning the separation technique.

**Figure 8:**
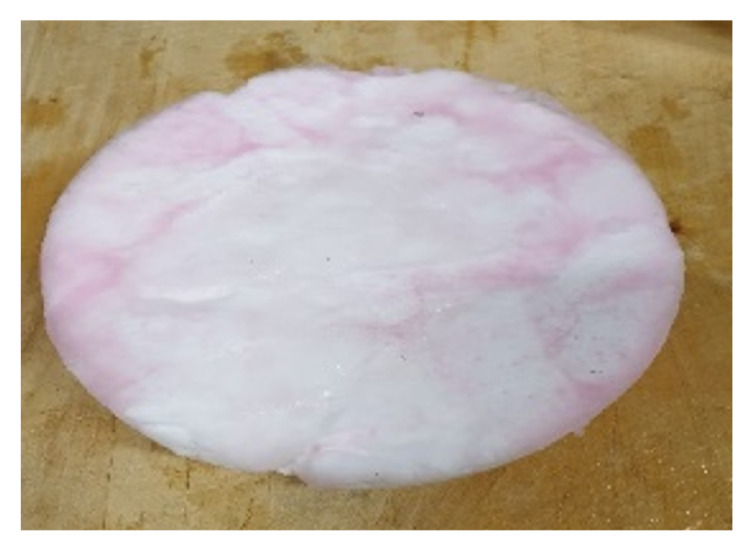
Hydrate pellet obtained from pressing after hydrate formation (at 37.5 bar and 3 °C) in the water–sucrose–betanin model solution.

Figure 8 depicts the hydrate pellet obtained via pressing from experiments conducted with the water-sucrose-betanin model solution. This figure visualizes the separation quality within the scope of this research, which clarifies the need for further optimization. Figure 3b,c present the concentrated water–sucrose–betanin model solution adhered to the hydrate structure. After pressing, parts of this concentrate are still visible on the hydrate pellet. In other studies pressing was used for the separation of hydrate and concentrate with promising results [13,21]. However, Claßen et al. (2020) [13] highlight that pressing should be performed under hydrate stable conditions for sugar contents above 30 °Brix. As this requires a high technical effort, the separation was not performed under hydrate stable conditions within the present research. Pressing outside of the stability range induced hydrate dissociation of the metastable hydrate. From the results of the water–sucrose–betanin model solution, in this research it has been shown that the drained liquid from the pressed hydrate is adhering concentrate with high sucrose and betanin content. During the investigation of fruit juices, it appeared that polyphenols, carotenoids, and vitamin C are not as much present in the drained liquid from the pressed hydrate as in the concentrate. The dilution that is induced by the pressing step contributes to this result. For this reason, in this research, the additional washing step that was suggested by Claßen et al. (2020) [13] was not realized, since no further dilution of adhering concentrate should take place. The inefficient separation is an issue that needs to be addressed to produce high-quality concentrates while using hydrate technology. Besides the separation technique, the water solubility of the analyzed compounds could influence the separation efficiency. Especially, carotenoids that are insoluble in water remain in the hydrate phase during pressing. For this reason, the continuous removal of hydrate crystals should take place before valuable fruit juice components could adhere to the hydrate and its porous structure. As an alternative, hydrate slurries could be produced. This approach is an advantage over bulky hydrate blocks, especially for continuous processes. The produced slurries could be separated by centrifugation or filtration.

## 5. Conclusions

This research had the main aim to investigate whether valuable ingredients of juices are accumulated in the concentrate or the hydrate during the concentration of fruit juices and model solutions while using gas hydrate technology. The presented results show, for the first time, that all examined substances can be mainly found in the concentrate. Only small amounts remain in the hydrate phase due to an inefficient separation technique. Therefore, improved processing could lead to concentrates of even higher quality. For an industrial application, the continuous removal of hydrate particles could enable a better separation. In future studies, either the mechanisms behind the possible incorporation of valuable juice compounds or quantitative effects on more specific phenols or carotenoids should be analyzed instead of total phenolics and total carotenoids. This would take different molecular structures and sizes into account. Besides, it might become important to characterize the inhibitory or promoting effects of certain compounds of liquid foodstuffs. Furthermore, the present study showed that a suitable operating point for hydrate formation is at 37.5 bar and 3 °C for concentrating a water–sucrose model solution from 10 °Brix to nearly 18 °Brix. Because reactor types, like stirred tanks, provide higher sugar concentrations than the used bubble column reactor, in the future it will be necessary to clarify what the most suitable reactor concept is.

## Figures and Tables

**Table 1:** An overview of the media used, indicating the substances considered, the experimental target, and the experimental repetition.

Medium	Analyzed Substances	Aim of Experiment	Hydrate Formation Repetitions
Model solution (water, sucrose)	Sucrose	Identification of working point.	2
Model solution (water, sucrose, betanin)	Sucrose Betanin	Evaluation of concentrate quality and preservation during hydrate formation. Visual assessment of separation quality by red/pink color of betanin.	3
Apple juice (clear)	Total phenolics Vitamin C	Evaluation of concentrate quality and preservation during hydrate formation.	3 for total phenolics 2 for vitamin C
Apple juice (cloudy)	Vitamin C	Evaluation of concentrate quality and preservation during hydrate formation.	2
Orange juice	Total phenolics Total carotenoids Vitamin C	Evaluation of concentrate quality and preservation during hydrate formation.	3 for total phenolics and total carotenoids 2 for vitamin C

## Supplementary Materials

The following are available online at https://www.mdpi.com/2304-8158/10/3/626/s1, Table S1: Absolute values for the contents of betanin [g/L], vitamin C [mg/L], total phenolics [mg/L] and total carotenoids [mg/L] for the concentrate (C), the drained liquid pressed from the hydrate (L) and the hydrate phase (H) within all analyzed media and the corresponding standard errors of the mean (SEM), Table S2: Standard errors of the mean (SEM) of sucrose measurements [° Brix] and the concentration yields [%] for the concentrate produced from the water-sucrose model solution by gas hydrate technology presented in Figure 4 and Figure 5, Table S3: Standard errors of the mean (SEM) of c/c${}_{0}$ for sucrose, betanin, vitamin C, total phenolics and total carotenoids for the concentrate (C), the drained liquid pressed from the hydrate (L) and the hydrate phase (H) within all analyzed media presented in Figure 6 and Figure 7.

## References

[1] Yahia E.M., de la Rosa L.A., Alvarez-Parrilla E., González-Aguilar G.A. (2010). The Contribution of Fruit and Vegetable Consumption to Human Health. Fruit and Vegetable Phytochemicals: Chemistry, Nutritional Value and Stability.

[2] Singh G.M., Micha R., Khatibzadeh S., Shi P., Lim S., Andrews K.G., Engell R.E., Ezzati M., Mozaffarian D. (2015). Global, Regional, and National Consumption of Sugar-Sweetened Beverages, Fruit Juices, and Milk: A Systematic Assessment of Beverage Intake in 187 Countries. PLoS ONE.

[3] Priyadarshini A., Priyadarshini A., Rajauria G., Tiwari B.K. (2018). Market Dimensions of the Fruit Juice Industry. Fruit Juices: Extraction, Composition, Quality and Analysis.

[4] A.I.J.N. (2019). Worldwide Consumption of Fruit Juice and Fruit Nectar in 2017 and 2018, by Region (in Millions of Liters) [Graph]. *Statista*. https://www.statista.com/statistics/421179/worldwide-consumption-of-fruit-juice-and-fruit-nectar-by-region/.

[5] A.I.J.N. (2019). Consumption of Fruit Juice and Fruit Nectar in Europe in 2017 and 2018, by Country (in Millions of Liters) [Graph]. *Statista*. https://www.statista.com/statistics/421405/fruit-juice-and-fruit-nectar-consumption-by-country-europe/.

[6] Adnan A., Mushtaq M., ul Islam T., Rajauria G., Tiwari B.K. (2018). Fruit Juice Concentrates. Fruit Juices: Extraction, Composition, Quality and Analysis.

[7] Taylor B., Ashurst P.R. (2016). Fruit and juice processing. Chemistry and Technology of Soft Drinks and Fruit Juices.

[8] Neves M.F., Trombin V.G., Marques V.N., Martinez L.F. (2020). Global orange juice market: A 16-year summary and opportunities for creating value. Trop. Plant Pathol..

[9] A.I.J.N. (2019). Konsum von Direktsaft und aus Konzentrat Gewonnenem Fruchtsaft in der Europäischen Union in den Jahren 2007 bis 2018 (in Millionen Liter) [Graph]. *Statista*. https://de.statista.com/statistik/daten/studie/388496/umfrage/konsum-von-direktsaft-und-aus-konzentrat-gewonnenem-fruchtsaft-in-der-eu/.

[10] A.I.J.N. (2015). Konsum von Direktsaft und aus Konzentrat Gewonnenem Fruchtsaft in Deutschland in den Jahren 2007 bis 2014 (in Millionen Liter) [Graph]. *Statista*. https://de.statista.com/statistik/daten/studie/398797/umfrage/konsum-von-direktsaft-und-aus-konzentrat-gewonnenem-fruchtsaft-in-deutschland/.

[11] Seidl P., Loekman S., Sardogan M., Voigt E., Claßen T., Ha J., Luzi G., Sevenich R., Agudo J.R., Rauh C. (2019). Food technological potentials of CO_2_ gas hydrate technology for the concentration of selected juices. High Press. Res..

[12] Claßen T., Seidl P., Loekman S., Gatternig B., Rauh C., Delgado A. (2019). Review on the food technological potentials of gas hydrate technology. Curr. Opin. Food Sci..

[13] Claßen T., Jaeger M., Loekman S., Gatternig B., Rauh C., Delgado A. (2020). Concentration of apple juice using CO_2_ gas hydrate technology to higher sugar contents. Innov. Food Sci. Emerg. Technol..

[14] Aman Z.M., Koh C.A. (2016). Interfacial phenomena in gas hydrate systems. Chem. Soc. Rev..

[15] Lee H.J., Lee J.D., Linga P., Englezos P., Kim Y.S., Lee M.S., Do Kim Y. (2010). Gas hydrate formation process for pre-combustion capture of carbon dioxide. Energy.

[16] Linga P., Kumar R., Lee J.D., Ripmeester J., Englezos P. (2010). A new apparatus to enhance the rate of gas hydrate formation: Application to capture of carbon dioxide. Int. J. Greenh. Gas Control.

[17] Babu P., Linga P., Kumar R., Englezos P. (2015). A review of the hydrate based gas separation (HBGS) process for carbon dioxide pre-combustion capture. Energy.

[18] Scondo A., Sinquin A. Effect of Additives on CO_2_ Capture From Simulated Flue Gas By Hydrates Formation in Emulsion. Proceedings of the 7th International Conference on Gas Hydrates (ICGH 2011).

[19] Kikkinides E.S., Yang R.T., Cho S.H. (1993). Concentration and Recovery of CO_2_ from Flue Gas by Pressure Swing Adsorption. Ind. Eng. Chem. Res..

[20] Peng X., Hu Y., Liu Y., Jin C., Lin H. (2010). Separation of ionic liquids from dilute aqueous solutions using the method based on CO_2_ hydrates. J. Nat. Gas Chem..

[21] Park K.N., Hong S.Y., Lee J.W., Kang K.C., Lee Y.C., Ha M.G., Lee J.D. (2011). A new apparatus for seawater desalination by gas hydrate process and removal characteristics of dissolved minerals (Na+, Mg_2_+, Ca_2_+, K+, B_2_+). Desalination.

[22] Babu P., Kumar R., Linga P. (2014). Unusual behavior of propane as a co-guest during hydrate formation in silica sand: Potential application to seawater desalination and carbon dioxide capture. Chem. Eng. Sci..

[23] Kang K.C., Linga P., Park K.N., Choi S.J., Lee J.D. (2014). Seawater desalination by gas hydrate process and removal characteristics of dissolved ions (Na+, K+, Mg2+, Ca2+, B3+, Cl−, SO42−). Desalination.

[24] Babu P., Nambiar A., He T., Karimi I.A., Lee J.D., Englezos P., Linga P. (2018). A Review of Clathrate Hydrate Based Desalination to Strengthen Energy-Water Nexus. ACS Sustain. Chem. Eng..

[25] Sloan E.D., Koh C.A. (2008). Clathrate Hydrates of Natural Gases.

[26] Sloan E.D. (2003). Fundamental principles and applications of natural gas hydrates. Nature.

[27] Koh C.A. (2002). Towards a fundamental understanding of natural gas hydrates. Chem. Soc. Rev..

[28] Li S., Shen Y., Liu D., Fan L., Tan Z., Zhang Z., Li W., Li W. (2015). Experimental study of concentration of tomato juice by CO_2_ hydrate formation. Chem. Ind. Chem. Eng. Q..

[29] Li S., Shen Y., Liu D., Fan L., Tan Z. (2015). Concentrating orange juice through CO_2_ clathrate hydrate technology. Chem. Eng. Res. Des..

[30] Purwanto Y.A., Oshita S., Seo Y., Kawagoe Y. (2001). Concentration of liquid foods by the use of gas hydrate. J. Food Eng..

[31] Safari S., Varaminian F. (2019). Study the kinetics and thermodynamics conditions for CO_2_ hydrate formation in orange juice concentration. Innov. Food Sci. Emerg. Technol..

[32] Loekman S., Claßen T., Seidl P., Luzi G., Gatternig B., Rauh C., Delgado A. Potential Application of Innovative Gas-Hydrate Technology in Fruit Juices Concentration Process. Proceedings of the 2019 World Congress on Advances in Nano, Bio, Robotics, and Energy (ANBRE19).

[33] Ghiasi M.M., Mohammadi A.H., Zendehboudi S. (2020). Clathrate hydrate based approach for concentration of sugar aqueous solution, orange juice, and tomato juice: Phase equilibrium modeling using a thermodynamic framework. Fluid Phase Equilibria.

[34] Chun M.K., Lee H. (1999). Phase Equilibria of Carbon Dioxide Hydrate System in the Presence of Sucrose, Glucose, and Fructose. J. Chem. Eng. Data.

[35] Carroll J. (2020). Natural Gas Hydrates: A Guide for Engineers.

[36] Sloan E.D. (2000). Clathrate hydrates: The other common solid water phase. Ind. Eng. Chem. Res..

[37] Jeffrey G.A., McMullan R.K., Cotton F.A. (1967). The clathrate hydrates. Progress in Inorganic Chemistry.

[38] Jeffrey G.A. (1984). Hydrate inclusion compounds. J. Incl. Phenom..

[39] Englezos P., Kalogerakis N., Dholabhai P., Bishnoi P. (1987). Kinetics of formation of methane and ethane gas hydrates. Chem. Eng. Sci..

[40] Shindo Y., Lund P.C., Fujioka Y., Komiyama H. (1993). Kinetics and mechanism of the formation of CO_2_ hydrate. Int. J. Chem. Kinet..

[41] Brown T.D., Taylor C.E., Bernardo M.P. (2010). Rapid Gas Hydrate Formation Processes: Will They Work?. Energies.

[42] Luo Y.T., Zhu J.H., Fan S.S., Chen G.J. (2007). Study on the kinetics of hydrate formation in a bubble column. Chem. Eng. Sci..

[43] Linga P., Daraboina N., Ripmeester J.A., Englezos P. (2012). Enhanced rate of gas hydrate formation in a fixed bed column filled with sand compared to a stirred vessel. Chem. Eng. Sci..

[44] Xu C.G., Li X.S., Lv Q.N., Chen Z.Y., Cai J. (2012). Hydrate-based CO_2_ (carbon dioxide) capture from IGCC (integrated gasification combined cycle) synthesis gas using bubble method with a set of visual equipment. Energy.

[45] Myre D., Macchi A., Servio P. Synthesis of CO_2_ Hydrates in a Slurry Bubble Column. Proceedings of the 7th International Conference on Gas Hydrates (ICGH 2011).

[46] Chernov A.A., Pil’nik A.A., Elistratov D.S., Mezentsev I.V., Meleshkin A.V., Bartashevich M.V., Vlasenko M.G. (2017). New hydrate formation methods in a liquid-gas medium. Sci. Rep..

[47] Andrés-Lacueva C., Medina-Remon A., Llorach R., Urpi-Sarda M., Khan N., Chiva-Blanch G., Zamora-Ros R., Rotches-Ribalta M., Lamuela-Raventós R.M., de la Rosa L.A., Alvarez-Parrilla E., González-Aguilar G.A. (2010). Phenolic Compounds: Chemistry and Occurrence in Fruits and Vegetables. Fruit and Vegetable Phytochemicals: Chemistry, Nutritional Value and Stability.

[48] Singleton V.L., Orthofer R., Lamuela-Raventós R.M. (1999). [14] Analysis of total phenols and other oxidation substrates and antioxidants by means of folin-ciocalteu reagent. Methods Enzymol..

[49] Yahia E.M., de Jesús Ornelas-Paz J., de la Rosa L.A., Alvarez-Parrilla E., González-Aguilar G.A. (2010). Chemistry, Stability, and Biological Actions of Carotenoids. Fruit and Vegetable Phytochemicals: Chemistry, Nutritional Value and Stability.

[50] DIN EN 12136:1997-12 Fruit and Vegetable Juices—Determination of Total Carotenoid Content and Individual Carotenoid Fractions; German Version EN 12136:1997. https://www.beuth.de/de/norm/din-en-12136/3572435.

[51] Rückemann H. (1980). Methoden zur Bestimmung von L-Ascorbinsäure mittels Hochleistungs-Flüssigchromatographie (HPLC). Z. Lebensm. Unters. Forsch..

[52] Sood S.P., Sartori L.E., Wittmer D.P., Haney W.G. (1976). High-pressure liquid chromatographic determination of ascorbic acid in selected foods and multivitamin products. Anal. Chem..

