# Supplementary material for: Concentrating Model Solutions and Fruit Juices Using CO2 Hydrate Technology and Its Quantitative Effect on Phenols, Carotenoids, Vitamin C and Betanin
Source: Foods. 2021 Mar 16;10(3):626. doi: 10.3390/foods10030626 (PMC7999093; doi:10.3390/foods10030626)
Supplement: Supplementary file 1 [file foods-10-00626-s001.pdf]

Table S1. Absolute values for the contents of betanin [g/L], vitamin C [mg/L], total phenolics [mg/L] and total carotenoids [mg/L] for the concentrate (C), the drained liquid pressed from the hydrate (L) and the hydrate phase (H) within all analyzed media and the corresponding standard errors of the mean (SEM).

Medium	Phase	Mean of the betanin content [g/L]	SEM [g/L]	Mean of the vitamin C content [mg/L]	SEM [mg/L]	Mean of total phenolics [mg/L]	SEM [mg/L]	Mean of total carotenoids [mg/L]	SEM [mg/L]
Model solution (water, sucrose, betanin)	C	1.65	0.10	-	-	-	-	-	-
	L	1.45	0.07	-	-	-	-	-	-
	H	0.53	0.05	-	-	-	-	-	-
Apple juice (clear)	C	-	-	1.02	0.04	609.99	20.24	-	-
	L	-	-	0.91	0.07	271.71	9.39	-	-
	H	-	-	0.25	0.03	72.07	9.76	-	-
Apple juice (cloudy)	C	-	-	235.07	44.64	-	-	-	-
	L	-	-	123.91	30.16	-	-	-	-
	H	-	-	31.76	11.58	-	-	-	-
Orange juice	C	-	-	528.40	0.67	771.27	120.33	7.34	0.19
	L	-	-	290.51	12.71	445.96	32.60	4.93	0.41
	H	-	-	4.71	1.81	164.93	11.29	3.72	0.07

Table S2. Standard errors of the mean (SEM) of sucrose measurements [° Brix] and the concentration yields [%] for the concentrate produced from the water-sucrose model solution by gas hydrate technology presented in Figure 4 and Figure 5.

Medium	Pressure [bar]	SEM for the sucrose measurements at 1 °C [°Brix]	SEM for the sucrose measurements at 3 °C [°Brix]	SEM for the sucrose measurements at 5 °C [°Brix]
Model solution (water, sucrose, betanin)	32.5	0.70	0.60	0.45
	37.5	0.30	0.20	0.30
	40	0.05	0.45	0.05

Medium	Pressure [bar]	SEM for the concentration yields at 1 °C [%]	SEM for the concentration yields at 3 °C [%]	SEM for the concentration yields at 5 °C [%]
Model solution (water, sucrose, betanin)	32.5	6.90	1.70	6.26
	37.5	0.55	0.30	2.43
	40	0.90	0.63	4.32

Table S3. Standard errors of the mean (SEM) of c/c_0 for sucrose, betanin, vitamin C, total phenolics and total carotenoids for the concentrate (C), the drained liquid pressed from the hydrate (L) and the hydrate phase (H) within all analyzed media presented in Figure 6 and Figure 7.

**Medium**	**Phase**	**SEM of c/c_0 for sucrose [-]**	**SEM of c/c_0 for betanin [-]**	**SEM of c/c_0 for vitamin C [-]**	**SEM of c/c_0 for total phenolics [-]**	**SEM of c/c_0 for total carotenoids [-]**
Model solution (water, sucrose, betanin)	C	0.114	0.061	-	-	-
	L	0.086	0.044	-	-	-
	H	0.039	0.032	-	-	-
Apple juice (clear)	C	-	-	0.046	0.128	-
	L	-	-	0.074	0.190	-
	H	-	-	0.035	0.103	-
Apple juice (cloudy)	C	-	-	0.001	-	-
	L	-	-	0.036	-	-
	H	-	-	0.098	-	-
Orange juice	C	-	-	0.016	0.259	0.011
	L	-	-	0.033	0.071	0.061
	H	-	-	0.006	0.026	0.003
